# Early Attentional Modulation by Working Memory Training in Young Adult ADHD Patients during a Risky Decision-Making Task

**DOI:** 10.3390/brainsci10010038

**Published:** 2020-01-09

**Authors:** Manon E. Jaquerod, Sarah K. Mesrobian, Alessandro E. P. Villa, Michel Bader, Alessandra Lintas

**Affiliations:** 1 NeuroHeuristic Research Group, HEC-Lausanne, University of Lausanne, Quartier UNIL-Chamberonne, 1015 Lausanne, Switzerland; manon.jaquerod@unil.ch (M.E.J.); smesrobian@neuristic.org (S.K.M.); Alessandro.Villa@unil.ch (A.E.P.V.); 2 University Service of Child and Adolescent Psychiatry, University Hospital of Lausanne, 1014 Lausanne, Switzerland; bader_m@bluewin.ch; 3 Faculty of Law, Criminal Justice and Public Administration, University of Lausanne, Quartier UNIL-Chamberonne, 1015 Lausanne, Switzerland

**Keywords:** working-memory training, selective attention, cognitive remediation, EEG, ERP, P1, P3b, N500, late posterior negative slow wave, late parietal negativity

## Abstract

**Background**: Working memory (WM) deficits and impaired decision making are among the characteristic symptoms of patients affected by attention deficit/hyperactivity disorder (ADHD). The inattention associated with the disorder is likely to be due to functional deficits of the neural networks inhibiting irrelevant sensory input. In the presence of unnecessary information, a good decisional process is impaired and ADHD patients tend to take risky decisions. This study is aimed to test the hypothesis that the level of difficulty of a WM training (WMT) is affecting the top-down modulation of the attentional processes in a probabilistic gambling task. **Methods**: Event-related potentials (ERP) triggered by the choice of the amount wagered in the gambling task were recorded, before and after WMT with a the dual *n*-back task, in young ADHD adults and matched controls. For each group of participants, randomly assigned individuals were requested to perform WMT with a fixed baseline level of difficulty. The remaining participants were trained with a performance-dependent adaptive *n*-level of difficulty. **Results**: We compared the ERP recordings before and after 20 days of WMT in each subgroup. The analysis was focused on the time windows with at least three recording sites showing differences before and after training, after Bonferroni correction (p<0.05). In ADHD, the P1 wave component was selectively affected at frontal sites and its shape was recovered close to controls’ only after adaptive training. In controls, the strongest contrast was observed at parietal level with a left hemispheric dominance at latencies near 900 ms, more after baseline than after adaptive training. **Conclusion**: Partial restoration of early selective attentional processes in ADHD patients might occur after WMT with a high cognitive load. Modified frontal sites’ activities might constitute a neural marker of this effect in a gambling task. In controls, conversely, an increase in late parietal negativity might rather be a marker of an increase in transfer effects to fluid intelligence.

## 1. Introduction

The information necessary for complex cognitive tasks, which require the expectation that a relevant stimulus is remembered, must be encoded and maintained in working memory (WM) with a prior selective attention that is necessary to ignore irrelevant information for further processing. Patients diagnosed with attention deficit/hyperactive disorder (ADHD) are characterized by poor WM, poor concentration, high impulsivity, tendency to excessive talking, impairement in maintaining focused attention and a multiple range of associated disorders [1,2,3,4]. Limited or untidy attentional resources in ADHD patients would reduce the anticipation of ensuing stimuli to be remembered and the amount of information that can be encoded [5,6]. Impaired selective attention processes during encoding information in WM and the resulting WM deficits have been observed in ADHD patients in association with altered functional connectivity of cortical and subcortical networks involving, in particular, the prefrontal cortex (PFC) [7,8,9,10]. Besides, neurophysiological evidence show that improvement in WM performance is achieved by invariant and distributed neuronal dynamics in the PFC [11].

A growing body of evidence shows that a few weeks of WM training for children and adults suffering from ADHD has positive behavioral and cognitive effects [12,13,14,15], Transfer effects reported after WM training [16,17] suggest that such training could be an alternative therapeutic approach to drugs for ADHD patients [18,19,20]. However, some comprehensive reviews and meta-analyses draw a more skeptical conclusion [21,22,23,24]: the training has a limited efficacy, the generalization and the duration of the effects are questionable, and the underlying neurophysiological processes remain unclear.

It is known that WM deficits are associated with impaired decision making in individuals with substance addictions and alcohol-dependency [25,26]. Risky decision making in an experimental task, the Iowa gambling task, is poorly performed by ADHD patients [27,28] and WM impairments characterizing ADHD were suggested to moderate the expression of risky decision-making in patients affected by this disorder [29,30,31]. Indeed, ADHD patients often choose riskier options with unfavorable outcomes in economic and financial settings [32,33]. More generally, substance use disorders, pathological gambling, and ADHD [26,34,35,36], as well as healthy participants charged with a high WM load [37], shared deficits in tasks associated with ventral prefrontal cortical dysfunction. On the one hand, the structural abnormalities observed in young adults with ADHD suggest complex audio-visual, motivational, and emotional dysfunctions [38]. The dual *n*-back task, on the other hand, is a WM training task in which the participants have to remember two independent sequences of audio-visual stimuli and must identify when an auditory or visual stimulus matches the one that appeared *n* trials back [39,40].

In the current work, we extend our previous study with EEG recordings, which showed differences in brain dynamics between controls and young adult patients with ADHD during the performance of a probabilistic gambling task [41]. Our working hypothesis is that WM training with the Dual *n*-back task is acting on a top-down modulation of the attentional processes with participation of prefrontal and parietal areas as sources of the efferent control signals. In the current study, we present new evidence that WM training affects selectively the activity of prefrontal cortex of young adult ADHD during a probabilistic gambling task. The P1-like waveform, elicited by the choice of the amount wagered, was restored in ADHD patients after WM training with the *adaptive level* variant of the Dual *n*-back task. We interpret this finding as an improvement of early higher-level mechanisms of attentional control in ADHD after adaptive training. In controls, the level of difficulty of WM training tended to affect late components of the event-related potentials (ERPs) mainly located at parietal areas.

## 2. Materials and Methods

### 2.1. Participants

This study was carried out in accordance with the latest version of the Declaration of Helsinki [42] and approved by the mandatory Ethics Committees requested by Swiss Federal Authorities, following the constitutional article (art. 118b Cst) of 8 March 2010 and the Federal Act involving Human Beings on 30 September 2011 (revised 1 January 2014). The ADHD patients were recruited either in the Psychiatric Department of the University Hospital of Lausanne or at a psychiatrist’s practice in collaboration with the Lausanne University Hospital after an initial screening appointment to ensure that they were fulfilling the criteria defined by the DSM-IV-TR for inattentive, hyperactive/impulsive or mixed subtypes [43]. Subjects with comorbid disorders and subjects taking medications were excluded from this study. We selected 65 young adults between 18 and 30 years old in the two groups of study, controls ($N_{CTRL}=37$) and ADHD patients ($N_{ADHD}=28$). Notice that control participants were recruited in the same age-range of the patients and with a similar social and educational background. Controls were screened prior to the experimental session to ensure that they would not report any disorder or exclusion criteria mentioned in the authorization released by the Ethics Committees. All participants were requested to fill French versions of the adult ADHD self-report scale (ASRS) and the Conners’ adult ADHD rating scales-self seport: screening version (CAARS-S:SV) [44,45,46] two weeks prior the begin of the protocol. All participants received a monetary compensation following the scale approved by the mandatory Ethics Committees (Commission cantonale d’ethique de la recherche sur l’être humain, code 101/12) requested by Swiss Federal Authorities.

### 2.2. Working Memory Task

**Figure 1:**
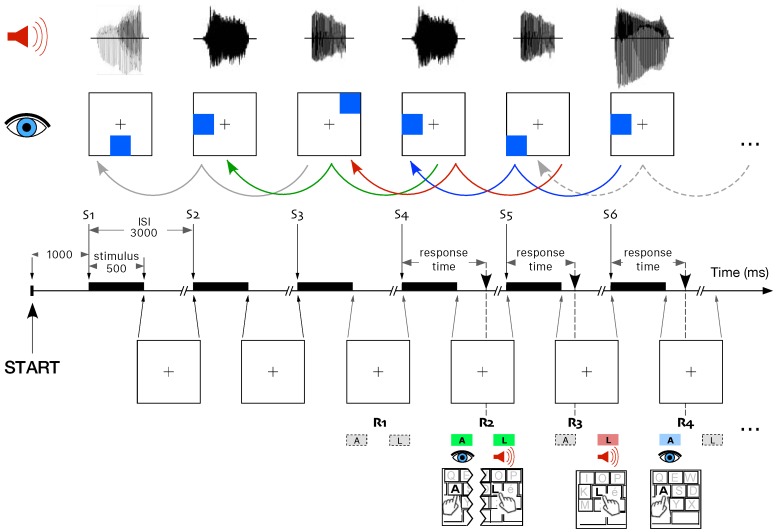
Level *n* = 2 of the dual *n*-back task. Each stimulus was composed by an auditory and a visual cue presented during 500 ms. This means the participants had to compare the third stimulus (S3) with the first one (S1), S4 with S2, S5 with S3, and so on. For the first correct response (R1), no stimuli matched those presented two trials back in time and no key press was requested. For R2, both auditory and visual stimuli matched the target (S4 identical to S2, green arrow)), such that both “A” and “L” key were pressed. For R3, only the auditory stimulus matched the target (red arrow) and only the “L” key was pressed. For R4, only the visual stimulus matched the target (blue arrow) and only the “A” key was pressed. Notice that in this example only correct responses are illustrated.

In this study the WM task consisted in two variants of the dual *n*-back task aimed at testing the divided attention [47,48]. Briefly, the task is the following. At each trial, an auditory and a visual cue were presented simultaneously during 500 ms, with an interstimulus interval (ISI) set to 3000 ms. The level of difficulty of the task is referred as *n*-back. The participants were asked to memorize the dual modality cues in order to compare the current auditory and visual stimuli with those presented *n*-trials back in time with the value *n* always the same for auditory and visual stimuli. In the conditions under which the current stimulus is not the same as the cue presented *n*-trials earlier, no response was requested by the participants. The participants had to press the “A” key for any visual stimulus matching the same stimulus presented *n*-trials back in time and/or the “L” key for any auditory stimulus matching the same stimulus presented *n*-trials back in time. If the participants did not respond within the fixed ISI, the trial was accounted as no response. Immediately after the response, a green light was switched on for correct response, otherwise a red light indicated a mistake. If “no response” was the correct choice, the green light switched on at the end of the ISI. In the case of baseline level, the difficulty of the task was set to *n* = 1. Figure 1 illustrates the dual *n*-back task at level *n* = 2 of difficulty.

In the case of adaptive level, the difficulty *n* of the task was adjusted as a function of the performance. The whole task consisted of 20 blocks of 20 + *n* trials with the same level of difficulty. An increase by 1 in the level of difficulty in the next block was triggered by a performance of less than three mistakes in each modality. With levels of difficulty higher than 1, a decrease by 1 in the level was triggered by five or more errors cumulated in any modality. The total duration of the working memory task was approximately half an hour.

### 2.3. Working Memory Training Protocol

In a pre-training session, at the laboratory, all participants played the adaptive version of the dual *n*-back task. At this session, the participants performed the WAIS-IV (Wechsler Adult Intellicence Scale-Fourth Edition) digit span subtest from the Wechsler adult intelligence scale, which requires participants to sequentially order the numbers (i.e., backward and forward digit span sequencing) presented by the examiner [49], the forward span of the Corsi block-tapping task, which is a visuospatial short-term memory task [50] and the attentional network test (ANT) [51]. The analysis of ANT will be presented in another paper. The WM training started the day after the pre-training session. At home, the participants played the dual *n*-back task by mean of an Internet remote connection to a server with protected access. The strict requirement was to complete at least 18 training sessions within a month. Randomly assigned participants in both controls and ADHD group were requested to perform a WM training either with a fixed baseline level of difficulty, i.e., dual 1-Back, or with a performance-dependent adaptive *n*-level of difficulty. A post-training session similar to the pre-training session was scheduled at the end of WM training [48]. All participants played the adaptive version of the dual *n*-back task at the post-training session. Please notice that all the analyses in this paper refer to the data acquired during the pre-training and the post-training sessions.

### 2.4. Probability Gambling Task

**Figure 2:**
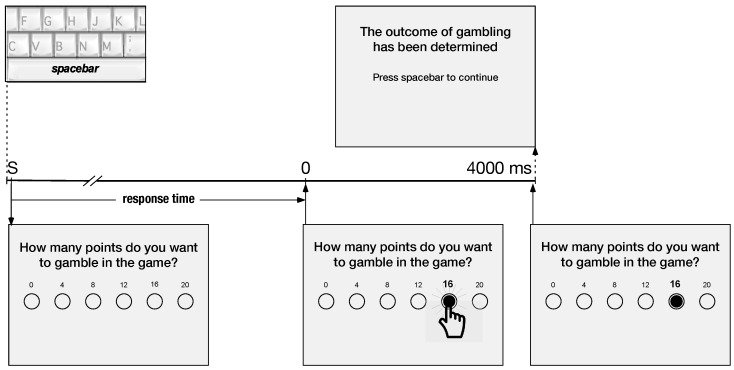
Probabilistic gambling task. A trial started when the participant pressed the spacebar (event S in the timeline), followed (20 milliseconds later) by a screen with a message request to select the gamble. This screen stayed on until a response was made by clicking on the selected value of gamble (event 0). The response time was determined by the interval between that message and the selection of gamble. This button click (event 0) was used as triggering event for the electrophysiological analysis. A fixed interval of 4000 ms followed until the end of the trial with the same screen and with the highlighted selected gamble.

The probability gambling task (PGT) used in this study was derived from a modified Gneezy–Potters’ task [48,52]. In summary, at the beginning of each trial an amount of 20 points was endowed to each participant. At each trial, the participant had to choose the amount wagered (as illustrated by Figure 2). The probability to win was set to 1/3, which meant a gain equal to 4× the gamble. In the event of a loss, at the end of the trial, the participant loses the entire amount wagered for that trial and keeps the rest of the initial endowment (which was always equal to 20). If the bet was equal to 16, then at the end of the trial the participant would receive 4 points in the event of a loss (i.e., 4=(20−16)) and 68 points in case of a win (i.e., =(20−16)+(4×16)). Notice that in this study the participant was just informed that the outcome of the bet was determined without any feedback on the amount earned, on the contrary of another study published elsewhere [41]. The click on the selected value of the bet with a mouse button is used as the triggering event for the electrophysiological analysis.

### 2.5. EEG Recording and Analyses

EEG was recorded using using 64 scalp Ag/AgCl active electrodes with impedances kept below 5 kΩ and referenced to the linked earlobes (ActiveTwo MARK II Biosemi EEG System, BioSemi B.V., Amsterdam, The Netherlands) mounted on a headcap (10/20 layout, NeuroSpec Quick Cap). Two pairs of bipolar electrodes were used to record ocular movements. EEG signals were recorded at 1024 Hz sampling frequency (24 bit resolution) and band-pass filtered between 0.05 Hz and 200 Hz. The selection of the amount to gamble (Figure 2, event 0) detected by a button-click was used to trigger the event-related potentials (ERPs). BrainVision Analyzer 2.0.4 (Brain Products, Gilching, Germany) was used for ERP preprocessing and removal of ocular artefacts by Infomax Independent Component Analysis (*ICA*) [53]. The ERP trials were cut into epochs starting 500 ms before and ending 1000 ms after the trigger. The interval of 500 ms prior to trigger onset was used for baseline correction. After removal of the trials characterized by easily identifiable artefacts, the epochs were visually inspected for contamination by residual minor artefacts. Artefact-free trials were filtered with lower cutoff at 0.1 Hz and upper cut-off at 30 Hz (−12dB/octave). Participants with less than 15 segments in any of the two recordings were excluded from this study. Analyses on the individual average whole-scalp ERP signals were performed with the software Cartool [54]. Those tests were applied with Bonferroni correction for the number of electrodes with a *p* value threshold at 0.05 [55].

## 3. Results

### 3.1. Participants’ Clinical Assessment

We used the R language and standard packages for the statistical analyses [56] and for each variable we report the values *m*, (M±SEM), corresponding to the median (*m*) and mean (*M*) ± standard error of the mean (SEM). Participant’s age for controls and ADHD was 22 years old (22.3±0.51) and 21 years old (22.1±0.71), respectively. The female-to-male gender ratio was 17:20 and 7:21 in controls and ADHD, respectively. The 2×2 contingency table showed no difference of gender ratio between the groups, $\chi^{2}\left(1,65\right)=2.17,p>0.05$.

A two-way analysis of variance, (group: controls, ADHD) × (gender: female, male), was run to assess ADHD symptoms. This analysis showed that normalized *T*-score values for CAARS-S:SV were always significantly higher for ADHD patients, such that it yielded a significant main effect for group, F(1,61)=35.98,p<0.001 for DSM-IV Inattentive Symptoms and F(1,61)=21.65,p<0.001 for the ADHD index. ADHD reported also higher values for ASRS than controls with a significant group effect, F(1,61)=11.19,p=0.001. The main effect of gender was always non-significant, F(1,61)=0.26,p>0.05, F(1,61)=2.18,p>0.05 and F(1,61)=0.004,p>0.05, for DSM-IV inattentive symptoms, ADHD index, and ASRS, respectively. The interaction effect was also non-significant F(1,61)=0.59,p>0.05, F(1,61)=1.94,p>0.05 and F(1,61)=1.12,p>0.05, for DSM-IV inattentive symptoms, ADHD index, and ASRS, respectively. In our previous paper [41] we have extensively analyzed and discussed the fact that there is a general agreement in the literature that there is no clear gender effect in young adult ADHD behavioral expression. For this reason we will not analyze further gender effects in this study, whose focus is the effect of the level of difficulty of the WM training protocol on the evoked brain activity.

### 3.2. Working Memory Performance

The effect of WM training was assessed by comparing the performance between the post- and pre-training sessions for the level *n* of difficulty achieved during the Dual *n*-Back task, the normalized score for the WAIS-IV digit span and the percentiles for the total score of the Corsi Block-Tapping Task (Table 1). A three-way analysis of variance, (group: controls, ADHD) × (WMT: pre-training, post-training) × (training level: baseline, adaptive) was carried out with a F(1,122) *F*-statistics for all main and interaction effects because all factors had two levels.

The ANOVA for the dual *n*-back task yielded a significant interaction between factors WMT and training level. A one-way analysis of variance for the pre-training and post-training sessions separately yielded a significant effect of the kind of training protocol (F(1,63)=7.70, p<0.01, and F(1,63)=19.35, p<0.001, respectively) on the average *n*-back level achieved by the participants. Another one-way analysis of variance for the baseline or the adaptive training protocol separately yielded a significant effect of the WM training (F(1,62)=15.20, p<0.001, and F(1,64)=94.25, p<0.001, respectively). This can be interpreted as some bias effect due to the initial random assignment of the participants to either the baseline or the adaptive training protocol. Table 1 shows that before WM training, the participants assigned to the baseline training protocol performed better than those assigned to the adaptive protocol (on average 2.20±0.12 vs. 1.90±0.06 and 2.10±0.13 vs. 1.84±0.11 for controls and ADHD, respectively). Despite this bias, the outcome of WM training was such that after being trained with the adaptive protocol both groups showed a better performance than being trained with the baseline protocol (on average 3.80±0.23 vs. 2.91±0.17 and 3.55±0.29 vs. 2.52±0.16 for controls and ADHD, respectively). This means that a one-month training of working memory had an effect on the outcome of the dual *n*-back task and that a training by the adaptive protocol produced a larger effect than baseline. Hence, the simple main effects on training level and WMT were truly significant by themselves, irrespective of the group of participants.

The WAIS-IV digit span showed no interaction between factors (Table 1), such that all significant simple main effects for factors *group*, *WMT* and *training level* can be considered as independent. This means that ADHD’s performance to this digit span sequencing test was poorer than in controls, and that Dual *n*-Back adapative training improved performance to WAIS-IV Digit Span irrespective of the group of participants. On the opposite, no significant effect was found for the visuospatial short-term memory assessed by the Corsi block-tapping task.

### 3.3. Probabilistic Gambling Task

The response time during the PGT, measured as indicated in Figure 2, decreased in all groups from the pre- to the post-training session, F(1,122)=18.65 (p<0.001), thus showing a significant main effect for factor *WMT*, irrespective of the training condition. In addition, Table 2 shows that the response time in controls was shorter than in ADHD, as revealed by the significant main effect of factor *group*. The WT training did not affect the total gains earned by all participants at the Probabilistic Gambling Task, irrespective of the group and the training condition. A Risk index RI=(HIR−−LIR)/(HIR+LIR) is calculated as a function of LIR, corresponding to low valued gambles (i.e., small amounts equal to 0, 4, or 8 points were gambled by the participant), and HIR, corresponding to high value gambles (i.e., the participant gambled 12, 16, or 20 points). The index RI is centralized such that a risk averse strategy is characteristic by RI≈−1, a risk neutral attitude by RI≈0 and a risky decision-making by RI≈1. It is interesting to notice that ANOVA shows the only significant main factor for Risk index is training level (Table 2). A two-way analysis of variance, (group: controls, ADHD) × *training level*: baseline, adaptive), was run for the pre- and post-training sessions separately. Before training, the two-way analysis of variance shows that the factor ttraining level was not significant (F(1,61)=3.37, p>0.05). On the contrary, after training the factor *training level* affected the Risk index (F(1,61)=5.40, p=0.023). In the baseline training condition, the RI increased on average by 0.07 and by 0.04 for controls and ADHD, respectively, from the pre- to the post-training session. This means that a WMT in the baseline condition tended to increase a risk taking attitude in both groups. Conversely, the adaptive training tended to increase a risky decision making in controls but in ADHD it tended to increase risk aversive attitude. However, *t*-test were not significant for each of these comparisons taken separately.

### 3.4. Event Related Potentials Triggered by Gambling Choice

**Figure 3:**
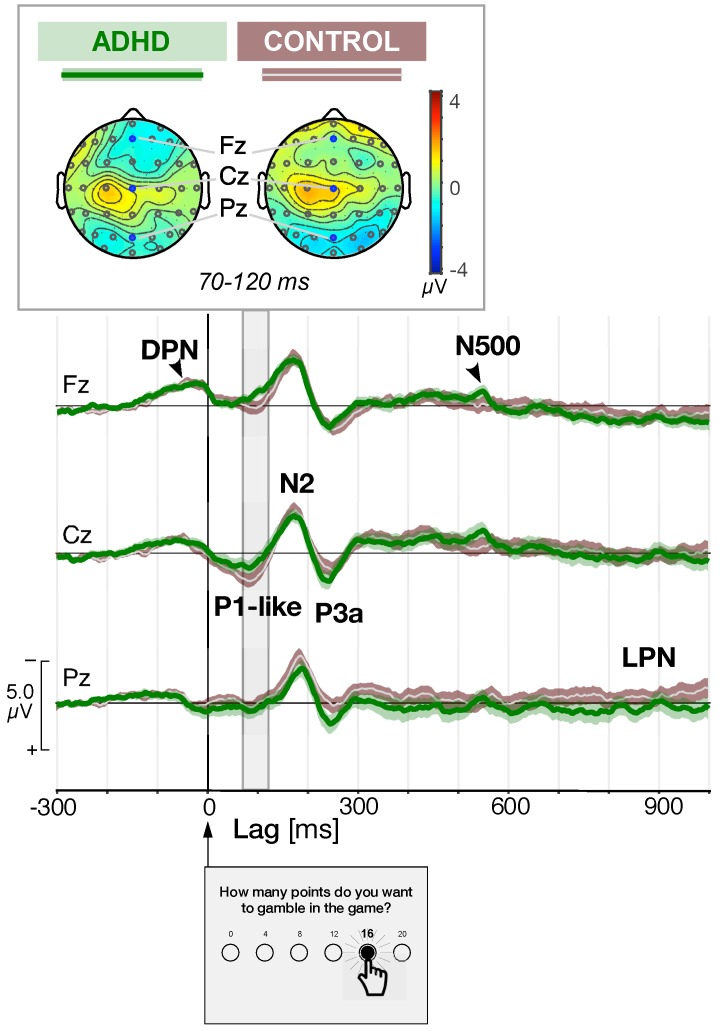
Grand average event-related potentials (ERPs) recorded before the working memory (WM) training at Fz, Cz and Pz sites triggered at lag 0, corresponding to button-click of the selected gamble, in attention deficit/hyperactivity disorder (ADHD) (N=28, green curves over light green shaded areas) and control participants (N=37, white curves over brown shaded areas) on a millisecond scale. The confidence interval (mean curve ± SEM) is shown by the shaded areas. We identified the decision preceding negativity (DPN), P1-like, N2, P3a, N500, and a late parietal negativity (LPN). Signal amplitude is scaled in microvolts (μV). The topographic maps on the top represent the distribution of the mean amplitude of the signal between 70 and 120 ms (estimated P1-like component) using a color-coded scale in μV.

In controls (N=37), the median number of epochs per participant was equal to 69 (63.8±2.6) and 71 (65.4±2.7) during the pre- and post-training sessions, respectively. In ADHD (N=28), we analyzed 48 (52.6±2.9) and 60 (58.3±2.6) epochs per participant during the pre- and post-training sessions, respectively. A three-way analysis of variance, (group: controls, ADHD) × (WMT: pre-training, post-training) × (*training level*: baseline, adaptive) yielded a significant main *group* effect, F(1,122)=11.17(p<0.01) for the number of epochs. This is due to the fact that EEG recordings of ADHD are always contaminated by more muscular artefacts than controls. It is important to notice that neither a main effect for the training level, F(1,122)=0.24(p>0.05), nor for the WMT, F(1,122)=1.52(p>0.05), was observed, thus validating the ERP analysis as a function of the WM training protocol in both groups of participants. Several positive and negative peaks were identified in the ERP grand averages waveforms in both control and ADHD participants before the training (Figure 3).

A negative readiness potential maximal at frontocentral electrodes, or decision preceding negativity (DPN), peaked at 40 ms before the trigger in both groups (Figure 3). After the trigger, we observed a positive wave component peaking at 90 ms in control participants (a P1-like component) corresponding to an early positive frontocentral deflection (Figure 3). Notice that in electrodes Fz and Cz, this P1-like component component was much less visible in ADHD participants, as confirmed by the topographic maps for the interval 70–120 ms, at the top of Figure 3. These topographic maps show also that this early positive component reaches its maximum at central electrodes, slightly lateralized on the left, and that ADHD patients are characterized by a stronger lateralization and a negative amplitude in frontal sites.

At all electrode sites, we observed a clear N2/P3 complex with N2 peaking at 180 ms and P3a peaking at about 250 ms. ADHD were characterized by a larger posterior P3 component than controls. The peak-to-peak amplitude between the N2 and P3a ERPs was measured for Pz, Cz and Fz. We ran a three-way ANOVA for factors (*group*: controls, ADHD), (WMT: pre-training, post-training) and (training level: baseline, adaptive) to determine any affect on the peak-to-peak amplitudes. We found no effect (p>0.05) of *group* with statistics F(1,122)=0.01, F(1,122)=1.03, and F(1,122)=0.37 for Pz, Cz and Fz, respectively. We found neither any effect (p>0.05) of WMT with statistics F(1,122)=0.03, F(1,122)=0.00, and F(1,122)=0.05 nor of *training level* with statistics F(1,122)=3.60, F(1,122)=0.00, and F(1,122)=0.23, for Pz, Cz and Fz, respectively. In Figure 3 we have also marked the N550 and the late parietal negativity (LPN). This latter component (LPN) is barely visible before training, in particular only in controls at site Pz in Figure 3. After training, LPN is very much affected and for this reason we have marked it already in this figure.

### 3.5. Effect of WM Training Condition on Differential Topographic Maps

**Figure 4:**
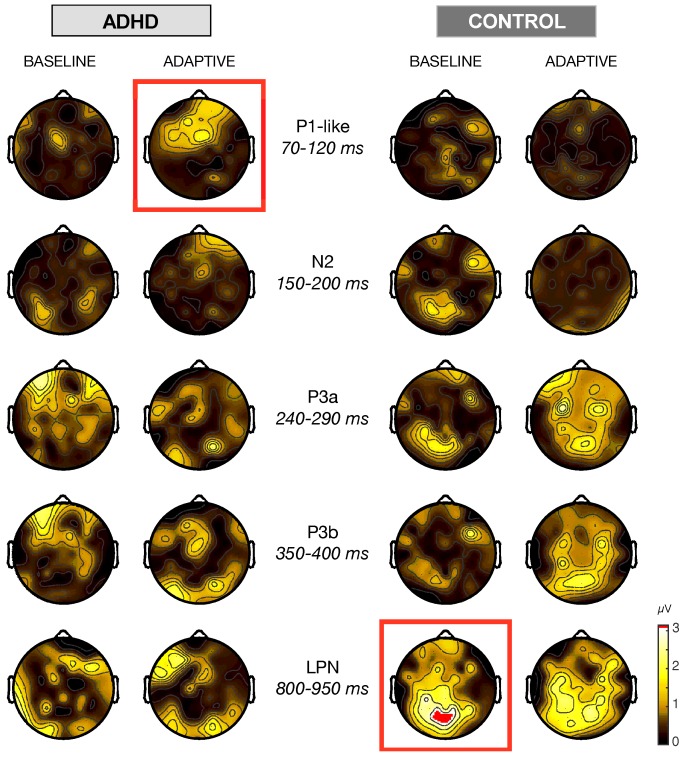
Differential head maps of the topographical distribution of the Grand-Average ERP amplitude (in μV) at post- minus pre-training sessions for ADHD and controls trained either by the baseline or adaptive protocol of the dual *n*-back task. ERPs were triggered by the choice of the selected gamble with the button-click. Differential head maps using a color-coded scale in μV are plotted for the five major ERP time windows. The red squares correspond to those head maps with significant Bonferroni-corrected *p*-values in the given time window, computed from paired t-tests on the individual average signals (see Figure 5).

At first, we compute the topographic head map distribution of the grand-average ERP amplitude (in μV) at post- and pre-training sessions for both subgroups of ADHD and controls, those who were trained in the baseline protocol (i.e., with the fixed level n=1 of the dual *n*-back task), and those with the adaptive protocol. After the ERP onset, corresponding to the choice of the selected gamble with the button-click, we determined five intervals of interest corresponding to the time course of the most relevant components observed in the ERPS. These wave components and their respective intervals were P1-like (70–120 ms), N2 (150–200 ms), P3a (240–290 ms), P3b (350–400 ms), and LPN (800–950 ms). All but LPN corresponded to time windows of 50 ms. The differential head maps were obtained with the topographic map for a specific time interval of the ERP at the post-training session minus the topographic map at the pre-training session for the same interval (Figure 4).

**Figure 5:**
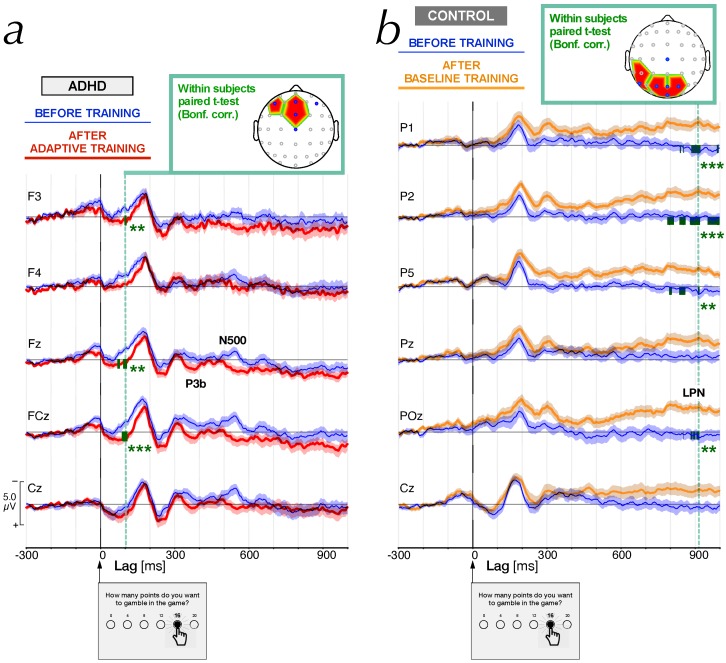
Grand average ERPs, triggered (at lag 0 ms) by the button-click at the time of the selection of the amount to gamble, recorded *before working memory training* (blue curves and shaded areas). The confidence interval (mean curve ± SEM) is shown by the shaded areas. The vertical scale represents the amplitude of the signal in μV and the lag is scaled in milliseconds. (**a**). Grand average ERPs at sites F3, F4, Fz, FCz and Cz sites (blue marks in the head map) for ADHD participants recorded after training with the adaptive level protocol (red curves and shaded areas) for the dual *n*-back task. Green ticks show the significant Bonferroni-corrected 1−p values computed from paired *t*-test on individual average ERP signal with significance p<0.05 (**) and p<0.01 (***). The panel at the top, shows a head map with the significant sites after Bonferroni correction (red areas around F3, Fz and FCz), at a latency of 100 ms (dashed green vertical line), corresponding to the P1-like component discussed in the text. (**b**). Grand average ERPs at sites P1, P2, P5, Pz, POz, and Cz sites (blue marks in the head map) for controls recorded after training with the baseline level protocol (orange curves and shaded areas), i.e., after the dual 1-back task. In this figure, the head map on the top shows the significant sites (in red areas around P1, P2, P5, CP3 and POz) at a latency of 912 ms (dashed green vertical line), corresponding to the slow negative wave component associated with the expectation of the gambling outcome.

Before the training, no difference was observed between averaged ERPs of either group assigned to adaptive and baseline training protocol. The most significant differential head maps were selected by applying a paired *t*-test with Bonferroni correction for the number of electrodes. We set a criterion of at least three electrode sites with a significant difference (p<0.05) during the very same time window within the interval of the selected wave component to define such significant differential head maps. The P1-like component was particularly affected in ADHD after the adapative training protocol (Figure 4, red square at first raw). Figure 5a shows that this component was increased in a significant way at frontocentral electrodes (F3, p<0.05; Fz, p<0.05; FCz, p<0.01). In this panel, notice that at site F4 the P1-like amplitude after training was also more positive than in the pre-training session, but the criterion of significance for the Bonferroni *t*-test correction was not reached. The grand average ERPS at site Cz is reported (Figure 5a) as a benchmark for a non-significant neighboring channel.

In the interval 150–200 ms, no training protocol produced any major effect on N2 head maps, neither for controls nor for ADHD. Notice that the differential head maps at P3a and P3b were very similar to each other in any of the subgroups. In controls, the maps showed increases in amplitudes at posterior sites, in particular, after adaptive training. Although these differences were significant for one or another channel, the criterion of three channels simultaneously significant for the paired *t*-test with Bonferroni correction was not reached. The late parietal negativity (LPN) was little affected in ADHD, but the differences in controls were large and mainly distributed over the parietal areas. In controls, Figure 5b shows that baseline training affected the ERPs already appear at wave components P3a and P3b, then disappeared at about 400 ms after the trigger onset. The maximal level of significance was observed at a lag near 900 ms, corresponding to LPN, where we observed significant Bonferroni-corrected *p* values at five posterior electrode sites (CP3, p<0.05; P1, p<0.01; P2, p<0.01; P5, p<0.05; POz, p<0.05) (Figure 4, red square at last raw). A similar but less significant effect was observed in controls after training with the adaptive dual *n*-back task.

## 4. Discussion

Working memory problems and impaired sustained attention are characteristic symptoms of ADHD [2,57]. Improvement of symptomatology by cognitive training and psychological interventions aimed to increase the correlation between sustained attention and arousal has been evalued several times in the recent past [58,59,60,61]. However, benefits for behavioral transfer effects to measures of fluid intelligence after several weeks of a computerized working memory treatment requiring high cognitive load could not be confirmed satisfactorily and raised questions about the controversial usefulness of such training [22,24,62]. The demand on cognitive processes is increased by the dual *n*-back task, which is a particular task aimed at challenging the divided attention by running visual and auditory modalities concurrently with the potential of WMT to compensate for a decline in executive functions [47,63]. In the current study, we have tested controls and ADHD patients who were trained during three weeks with the Dual *n*-Back Task. We included two subgroups, from controls and ADHD, who were trained with a non adaptive version of the task-with a fixed level of difficulty set to 1, that is a dual 1-back task (the *baseline* protocol).

We found evidence that WMT, irrespective of *baseline* or adaptive protocol, improved the score of the dual *n*-back task played by the participants at the post-training in comparison with the pre-training session, in agreement with past studies using fewer sessions of WMT [39]. Our training protocol lasted 20 days, a duration comparable with other studies reported in the literature using Dual *n*-Back Task [64]. In addition to increase in dual *n*-back scores in controls, we observed that WMT improved performance in the WAIS-IV Digit Span in agreement with previous studies [39,65,66]. It is important to notice that ADHD patients and controls are reliably differentiated by the WAIS-IV [67]. The novelty in our study is that for the first time, to our best knowledge, we report that ADHD patients improved their performance in the WAIS-IV Digit Span after a WM training protocol. After training, ADHD could perform at the same level of controls before training. However, measurement of the visuospatial working memory by the Corsi block-tapping task did not show any significant difference between controls and ADHD neither before nor after training. This finding is in agreement with the observation that visuospatial working memory is not specifically impaired in ADHD [68,69,70] and that dual *n*-back task is a working memory task affecting circuits other than those involved in visuospatial processing [66,71,72,73,74].

Before training, the results of the probability gambling task did not show any difference between ADHD and controls with respect to the total gain and risk index, in line with our previous study [41]. The analysis of the response time confirmed that ADHD responded at a significant slower pace than controls already before WMT, as previously reported [41]. However, training in both baseline and adaptive conditions provoked a faster reaction in both groups with similar magnitude, thus suggesting a similar process for an increase in the capacity to handle divided attentional stimuli in both ADHD and controls. The WM training failed to affect the total gains, but in the baseline condition it revealed a tendency to increase a risk taking attitude in both groups, matching our previous observation along the same line [75]. Controls tended to increase a risky decision making also after the adaptive training, somehow like after baseline training. On the contrary, after adaptive training, ADHD tended to decrease risk-taking attitude. This result suggests that improved divided attentional processes in both groups and opposite risk-taking behavior are elicited by a high cognitive load generated by the adaptive dual *n*-back task. An interpretation of this result is that these processes are controlled by different pathways, in agreement with literature on the behavioral deficits of ADHD patients [76,77,78,79,80].

Before the onset of the ERP trigger, we observed a negative readiness potential maximal at frontocentral electrodes, or decision preceding negativity (DPN), consistent with the literature and unaffected by WMT [81]. The ERPs following the gamble selection in the Probability Gambling Task [52] are characterized by several wave components [82,83,84,85]. Few studies analyzed the ERPs in adult ADHD and showed that N2, P3a, P3b, feed-back related negativity and N400-like components distinguish ADHD and controls in association with the evaluation of the reward outcome [28,86] and with the emotional feelings generated by risk-taking attitude [41]. In our current paper, we did not observe group effects (i.e., differences between controls and ADHD) on the measurements of PGT due to the working memory training, other than on the response times (Table 2). Our finding is in agreement with the observation that working memory training in general improves processing speed and attention performance [87] independently of the transfer to fluid intelligence [24,62,79] and that inattentive symptoms in ADHD are not associated with fluid intelligence [78,79,88] Hence, we do not discuss further the gambling task-related ERP components but P1 and LPN, the wave components which appeared to be the most affected by dual *n*-back task working memory training.

We observed a very significant effect of adaptive training on the P1-like wave component in the ADHD group. It is known that P1/N1 early sensory ERP components tend to be attenuated in ADHD patients [89,90,91,92] and our observations before training confirm those studies. Attentional modulation progress along the build-up of the ensuing P1-like [93,94]. The attenuation observed in ADHD can be interpreted following the perceptual load theory in selective attention [95,96]. In the current study, the P1-like wave is triggered by the button-click on the selected gamble. ADHD participants might have a degree of perceptual overload when facing the decision to invest, thus impairing their attentional resources as revealed by the attenuated P1-like. A WMT during three weeks with the adaptive training protocol of the dual *n*-back task generates a sustained high cognitive load on divided attention. The particular characteristic of this task is that the working memory capacity is solicited by the number of objects to be memorized and by the cross-modal features associated with the stimuli. P1 was found to likely reflect spatially based information shared by the auditory attention and visual memory systems that do not have to be mutually recruited in situations involving cross-modal tasks [97,98] and sensitive to the number of objects rather to the number of features to be memorized [99]. This is also in agreement with our finding, mentioned before, of an improvement in the score of the WAIS-IV digit span after WMT. The puzzling finding of an increase in P1-like wave amplitude in ADHD after the adaptive training, with restoration of a waveform similar to controls, is in favor of the hypothesis of an improvement of early higher-level mechanisms of attentional control in ADHD after adaptive training. The topological maps have clearly located this change of activity at the level of the prefrontal cortical areas. The P1 wave has been associated with an inhibitory feedback wave from “higher” cortical areas acting as an inhibitory filter to control feedforward sensory processes [100]. A change in P1 might be related to a change in the early modulation of attention, such to improve the sensory-perceptual level of processing that is necessary to improve the decisional process. There is evidence that modulation of neural activity by selective attention may occur at the subcortical level [101], where inhibitory gating mechanisms take place [102,103,104]. Then, an increase of the P1-like wave, in our paradigm, could be associated with a more effective processing of the decision due to a greater inhibition of potentially competing and task irrelevant networks.

The last ERP component strongly affected by WMT is the late parietal negative slow wave, whose amplitude was selectively increased in controls after both training level conditions. This wave started approximately 700 ms after the onset and extended for several hundreds of milliseconds. The topographic distribution of this wave is clearly parietal-posterior and with a left hemispheric dominance at latencies near 900 ms. In the literature, it is interesting to note that LPN has been observed as a neural marker related to the transfer of cross-modal associated information in working memory, [105,106] with memory tasks that required continued evaluation of contextual information [107,108,109] and with memory tasks that required high demands on action monitoring in presence of conflictual response options [110]. Before training, ERPs recorded in both groups did not show a relevant presence of LPN in the PGT. This suggests that WMT is a necessary condition to let appear LPN. Moreover, ADHD patients did not show any effect of WMT on LPN. If we consider all these observations together we may raise the hypothesis that WMT with the dual *n*-back task is able to generate a transfer effect in the PGT [111,112], as revealed by the LPN associated with a continued evaluation of contextual information in our PGT. This effect is strong in controls, but it is absent in our ADHD group, thus suggesting that the kind of WMT performed by our group of patients only confers benefits for those tasks that were trained [15,62,113]. We could speculate that a deficit in transfer effects associated with WM training could be associated with the abnormal parietal brain function observed in ADHD [114].

## 5. Conclusions

In conclusion, we have confirmed that working memory training produces cognitive effects for the task that was trained in both controls and ADHD patients. In particular, improvement in early attentional processes in ADHD is likely to be the most beneficial effect of WMT with the dual *n*-back task if the training required a high cognitive effort for divided attention, such as in the case of the adaptive condition. Transfer effects to fluid intelligence occurring only in controls might be associated with the development of a late parietal negativity elicited by a risky decision-making task.

## Figures and Tables

**Table 1:** Pre and post-training performance (median, mean, and SEM)to the memory tasks and results of the three-way analysis of variance (ANOVA).

	Group:	Controls			ADHD			*ANOVA*		
	Level:	Baseline	Adaptive		Baseline	Adaptive		*Effect*	F(1,122)	**Pr(>F)**
**Sample size (*N*)**		18	19		14	14				
**Dual*n*-Back level**								group:	2.76	>0.05
								level:	7.67	0.006 **
	*pre-training*	2.12	1.95		1.97	1.80		WMT:	101.2	<0.001 ***
		2.20 (0.12)	1.90 (0.06)		2.10 (0.13)	1.84 (0.11)		group×level:	0.13	>0.05
WMT:								group×WMT:	1.06	>0.05
	*post-training*	2.77	3.95		2.45	3.33		level×WMT:	26.4	<0.001 ***
		2.91 (0.17)	3.80 (0.23)		2.52 (0.16)	3.55 (0.29)		group×level×WMT:	0.04	>0.05
**WAIS-IV digit span (*normalized score*)**								group:	11.28	0.001 **
								level:	14.99	<0.001 ***
	*pre-training*	13.50	11.00		10.50	10.00		WMT:	6.95	0.009 **
		13.67 (0.68)	11.11 (0.31)		11.14 (0.94)	9.86 (0.72)		group×level:	2.22	>0.05
WMT:								group×WMT:	0.11	>0.05
	*post-training*	15.50	13.00		13.00	11.50		level×WMT:	0.03	>0.05
		14.83 (0.62)	12.26 (0.55)		12.43 (1.04)	11.57 (0.84)		group×level×WMT:	0.05	>0.05
**Corsi block-tapping task (*percentiles*)**								group:	0.06	>0.05
								level:	0.00	>0.05
	*pre-training*	90.0	80.0		70.0	80.0		WMT:	0.80	>0.05
		78.3 (5.1)	72.9 (4.9)		60.7 (7.4)	67.1 (6.8)		group×level:	0.00	>0.05
WMT:								group×WMT:	3.56	>0.05
	*post-training*	80.0	80.0		85.0	80.0		level×WMT:	0.00	>0.05
		78.9 (4.6)	69.0 (4.9)		70.4 (8.0)	75.4 (4.7)		group×level×WMT:	0.00	>0.05

**: p<0.01; ***: p<0.001.

**Table 2:** Pre and post-training performance (median, mean and SEM) during the probabilistic gambling task and results of the three-way analysis of variance (ANOVA).

	*Group:*	Controls			ADHD			*ANOVA*		
	*Level:*	Baseline	Adaptive		Baseline	Adaptive		*Effect*	F(1,122)	Pr(>F)
**Sample size (*N*)**		18	19		14	14				
**Response time (*ms*)**								group:	5.26	0.024 *
								level:	1.4	>0.05
	*pre-training*	914	1289		1305	1332		WMT:	18.65	<0.001 ***
		1199 (155)	1396 (203)		1542 (192)	1573 (233)		group×level:	0.01	>0.05
WMT:								group×WMT:	1.40	>0.05
	*post-training*	738	812		926	996		level×WMT:	0.01	>0.05
		764 (73)	826 (79)		942 (107)	1201 (217)		group×level×WMT:	0.61	>0.05
**Total Gains (*points*)**								group:	2.24	>0.05
								level:	1.17	>0.05
	*pre-training*	1886	1868		1812	1878		WMT:	0.05	>0.05
		1947 (48)	1875 (39)		1853 (46)	1902 (45)		group×level:	3.79	>0.05
WMT:								group×WMT:	0.55	>0.05
	*post-training*	1890	1868		1848	1806		level×WMT:	0.36	>0.05
		2010 (92)	1850 (58)		1809 (52)	1857 (65)		group×level×WMT:	0.27	>0.05
**Risk index**								group:	1.14	>0.05
								level:	8.68	0.004 **
	*pre-training*	0.18	−0.22		0.15	−0.08		WMT:	0.29	>0.05
		0.18 (0.12)	−0.15 (0.09)		−0.02 (0.14)	−0.09 (0.12)		group×level:	1.20	>0.05
WMT:								group×WMT:	0.10	>0.05
	*post-training*	0.18	−0.18		0.16	−0.04		level×WMT:	0.12	>0.05
		0.25 (0.14)	−0.09 (0.09)		0.06 (0.15)	−0.14 (0.10)		group×level×WMT:	0.04	>0.05

*: p<0.05; **: p<0.01; ***: p<0.001.
